# Recent Insights and Multifactorial Applications of Carbon Nanotubes

**DOI:** 10.3390/mi12121502

**Published:** 2021-11-30

**Authors:** Muthu Thiruvengadam, Govindasamy Rajakumar, Venkata Swetha, Mohammad Azam Ansari, Saad Alghamdi, Mazen Almehmadi, Mustafa Halawi, Lakshmanan Kungumadevi, Vaishnavi Raja, Sulthana Sabura Sarbudeen, Saranya Madhavan, Maksim Rebezov, Mohammad Ali Shariati, Alexandr Sviderskiy, Konstantin Bogonosov

**Affiliations:** 1 Department of Crop Science, College of Sanghuh Life Science, Konkuk University, Seoul 05029, Korea; muthu@konkuk.ac.kr; 2 Collaborative Innovation Center for Advanced Organic Chemical Materials Co-Constructed by the Province and Ministry, Ministry of Education Key Laboratory for the Synthesis and Application of Organic Functional Molecules, College of Chemistry and Chemical Engineering, Hubei University, Wuhan 430062, China; microlabsraj@gmail.com; 3 Annamacharya Institute of Technology & Sciences, Tirupati 517520, India; swethanadhphysics@gmail.com; 4 Department of Epidemic Disease Research, Institute for Research and Medical Consultations (IRMC), Imam Abdulrahman Bin Faisal University, Dammam 31441, Saudi Arabia; maansari@iau.edu.sa; 5 Laboratory Medicine Department, Faculty of Applied Medical Sciences, Umm Al-Qura University, Makkah 24382, Saudi Arabia; ssalghamdi@uqu.edu.sa; 6 Department of Clinical Laboratory Sciences, College of Applied Medical Sciences, Taif University, P.O. Box 11099, Taif 21944, Saudi Arabia; dr.mazen.ma@gmail.com; 7 Medical Laboratory Technology, Applied Medical Sciences College, Jazan University, Jazan 45142, Saudi Arabia; halawim@jazanu.edu.sa; 8 Department of Physics, Mother Teresa Women’s University, Kodaikanal 624101, India; sivarivudevi@gmail.com (L.K.); vaishnavitr1995@gmail.com (V.R.); cvssarbudeen@gmail.com (S.S.S.); 9 Department of Chemistry, D.K.M. College for Women, Vellore 632001, India; saranyamadhavan07@gmail.com; 10 Research Department, K.G. Razumovsky Moscow State University of Technologies and Management (The First Cossack University), 73, Zemlyanoy Val St., 109004 Moscow, Russia; rebezov@yandex.ru (M.R.); katsostud@mail.ru (K.B.); 11 Prokhorov General Physics Institute of the Russian Academy of Science, 38 Vavilova Str., 119991 Moscow, Russia; 12 Faculty of Engineering and Technology, Innovative University of Eurasia, 45 Lomov St., Pavlodar 140000, Kazakhstan; kafedrains@ya.ru

**Keywords:** nanomaterials, methodologies, carbon nanotubes, applications

## Abstract

Nanotechnology has undergone significant development in recent years, particularly in the fabrication of sensors with a wide range of applications. The backbone of nanotechnology is nanostructures, which are determined on a nanoscale. Nanoparticles are abundant throughout the universe and are thought to be essential building components in the process of planet creation. Nanotechnology is generally concerned with structures that are between 1 and 100 nm in at least one dimension and involves the production of materials or electronics that are that small. Carbon nanotubes (CNTs) are carbon-based nanomaterials that have the structure of tubes. Carbon nanotubes are often referred to as the kings of nanomaterials. The diameter of carbon is determined in nanometers. They are formed from graphite sheets and are available in a variety of colors. Carbon nanotubes have a number of characteristics, including high flexibility, good thermal conductivity, low density, and chemical stability. Carbon nanotubes have played an important part in nanotechnology, semiconductors, optical and other branches of materials engineering owing to their remarkable features. Several of the applications addressed in this review have already been developed and used to benefit people worldwide. CNTs have been discussed in several domains, including industry, construction, adsorption, sensors, silicon chips, water purifiers, and biomedical uses, to show many treatments such as injecting CNTs into kidney cancers in rats, drug delivery, and directing a near-infrared laser at the cancers. With the orderly development of research in this field, additional therapeutic modalities will be identified, mainly for dispersion and densification techniques and targeted drug delivery systems for managing and curing posterior cortical atrophy. This review discusses the characteristics of carbon nanotubes as well as therapeutic applications such as medical diagnostics and drug delivery.

## 1. Introduction

Nanoscience is an interdisciplinary field of study encompassing but not limited to physics, chemistry, biology, medicine, engineering, and materials science. Carbon nanotubes (CNTs) are nanostructured carbon allotropes with a larger than 1,000,000 length-to-diameter ratio [1]. Numerous nanotubes were synthesized through laser ablation, arc discharge, and chemical vapor deposition techniques [2]. Carbon nanotubes are the most powerful material known to humanity [3]. The greatest tensile strength of a carbon nanotube was measured to be 63 GPa, approximately 50 times that reported by Takakura et al. [3]. Even the tiniest carbon nanotubes have the strength of several GPa [3]. Carbon nanotubes are beneficial for various uses, including electrochemical supercapacitors, transistors, field-emitting devices, nanoprobes, sensors, composite materials, energy storage, hydrogen storage, and templates [4]. Carbon nanotubes were initially used to reinforce a range of structural materials used in optics, plastics, electronics, and other nanotechnology areas [5]. They have been used in pharmacy and medication delivery systems in therapies since the early twenty-first century. Owing to their high surface area, outstanding chemical stability, and rich electronic polyaromatic structure, carbon nanotubes can adsorb or combine a wide variety of medical substances, including medications, proteins, antibodies, DNA, and enzymes [6].

Carbon nanotubes are closed or open-ended cylinders made of one or more graphene layers (single-walled or multi-wall carbon nanotubes) [7]. All carbon atoms in useful carbon nanotubes are connected in a hexagonal lattice except at their ends, whereas faults in mass-produced carbon nanotubes include pentagons, heptagons, and other sidewalls imperfections, which often diminish beneficial qualities [8]. Sp^2^ carbon hybridization results in a layered structure with weak out-of-plane bonding and solid in-plane boundaries of the Van der Waals type. CNTs are typically classified as single-wall carbon nanotubes (SWCNTs), double-wall carbon nanotubes (DWCNTs), and multi-wall carbon nanotubes (MWCNTs) [9]. SWCNTs are a single cylindrical carbon sheet with a diameter ranging from 0.4 to 2 nm, depending on the temperature used to produce them. It was discovered that the higher the growing temperature, the smaller the diameter of the carbon nanotubes [6]. SWCNTs can be configured in an armchair, zigzag, chiral, or helical fashion [10]. Two structural models, the Doll model from Russia and the Parchment model from France, are capable of forming multi-wallet carbon nanotubes. When a CNT has another nanotube inside, and the diameter of the outer nanotube is greater than the diameter of the thinner nanotubes, which is referred to as the Russian Doll model. However, on the other hand, the Parchment model occurs when a single graphene sheet is wrapped several times around itself, similar to a rolled-up paper scroll [11]. Thus, in a relatively short period of time, scientists from various fields have developed an interest in carbon nanotubes. They may be significant antioxidants with the ability to protect health and prevent disease [12].

They exhibit intriguing features, including a high aspect ratio, ultra-lightweight construction, high strength, high thermal conductivity, and electrical properties spanning metal to semiconductor. Carbon nanotubes can be synthesized using chemical vapor deposition, or plasma-enhanced chemical vapor deposition (CVD) [13], plasma or arc discharge evaporation [14], laser ablation [15], thermal synthesis [16]. These processes all include the input of energy to the carbon source, resulting in fragments (groups or single carbon atoms) that can be recombined to form CNT. Electricity from the arc discharge, heat from the CVD furnace (about 900 °C), or high-intensity laser light may be used as the energy source [17]. Nanotube-based electric nanosensors permit biological monitoring without the need for labels. A protein microarray technique using carbon nanotube surface-enhanced Raman spectroscopy with detection sensitivity as low as 1 fmol/L is possible. Compared to in vitro toxicology studies, highly water-soluble and serum-stable nanotubes are biocompatible, nontoxic, and possibly useful for biomedical applications. Nanotubes aggregate throughout the reticuloendothelial system (RES), including the liver and spleen, after intravenous therapy, with biodistributions varied with the functionalization and, possibly, size of nanotubes. If nanotubes are fully functionalized, they can be removed mostly through the biliary system in feces. In vitro and in vivo studies have shown that carbon nanotubes may transport small interfering RNA (siRNA), paclitaxel, and doxorubicin [18].

**Figure 1:**
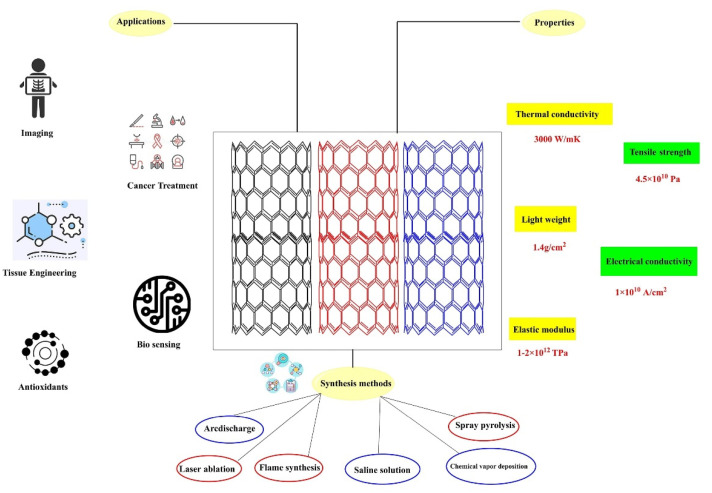
An overview of properties of CNTs and with synthetic and transdermal applications.

Carbon nanotubes (CNTs) have sparked significant interest in materials science due to their unique structure and magical properties. CNTs are increasingly being used in novel medication delivery methods, as well as bioimaging and biosensing. Recent efforts to functionalize carbon nanotubes have resulted in the production of biocompatible and water-soluble CNTs that are excellent for future cancer treatments with minimal pharmaceutical dosages, high therapeutic efficacy, and few side effects. This review covers the most recent breakthroughs in CNT modification strategies (with inorganic nanoparticles, small molecules, etc.) after a brief introduction to CNT toxicity [19]. By improving the synthesis process and studying the applications, the quality or quantity of material generated can be increased. Applications include biomaterials, composite materials, electronics, electromagnetic devices, optics, and biomedical devices. In this quick analysis, we will discuss the various applications of carbon nanotubes (Figure 1).

## 2. Review of the Carbon Nanotube Construction

Mohajerani et al. evaluated the unique features of nanostructured materials that have sparked attention in industry and research [20]. Meanwhile, the amazing qualities of carbon nanotubes have demonstrated the advancement of materials employed in civil constructions, the increasing abilities of modern sciences in the domains of structural and environmental behavior, as well as their high potential as newly found materials. We explored the application of nanoscience technology to the field of building structures, namely CNTs and their varieties, and will evaluate various distinctive characteristics of this nanostructure in construction engineering. They had no idea that the introduction of nanoscience into many disciplines of construction study would result in the emergence of a new world of science for humanity. Adhikary et al. investigated the effect of MWCNT dispersions on the structure of newly formed crystal hydrates in autoclaved cellular concrete’s interporous walls [21]. Microstructure and X-ray phase analysis of calcium hydro silicates formed during autoclave treatment of cellular silicate concrete modified with multiwalled carbon nanotubes revealed changes in the morphological features of new formations and the formation of calcium hydro silicates with enhanced basicity, resulting in an improvement in the physical and technical characteristics of autoclaved cellular concrete.

Saptarshi et al. suggest that nanoscience and nanotechnology hold immense promise for engineering the characteristics of materials at the atomic and molecular levels [22]. Not only did this transcend various limits inherent in conventional materials, but it also considerably boosted their mechanical, physical, and chemical capabilities. Carbon nanotubes could be used to change or increase the qualities of existing building materials such as concrete and steel in order to create high-performance, multipurpose, and ideal (high strength, flexibility, crack-free, and durable) building materials. Laukaitis et al. demonstrated that autoclaved aerated concrete containing carbon fiber absorbs electromagnetic waves by up to –30 dB in the frequency range of 2 to 18 GHz [23]. According to our research, adding 6% multiwalled carbon nanotubes to the silicate coating results in the silicate binding matrix being structured, producing a dense, strong, and lasting coating capable of absorbing electromagnetic radiation affecting buildings and facilities.

**Figure 2:**
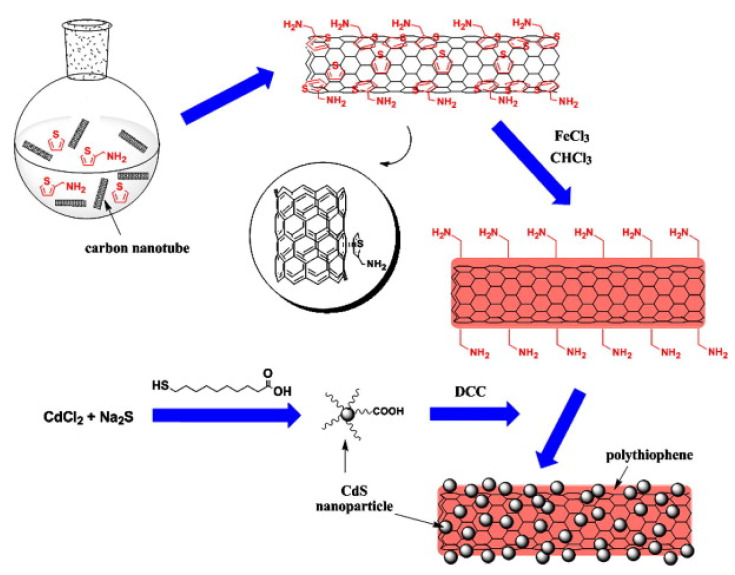
Schematic representation of decorating multiwalled carbon nanotubes with cadmium sulfide nanoparticles using in situ polymerized PTh serving as an inter-linker. (Reprinted with permission from [28]. Copyright 2010, Feng et al.).

The advancement of cost-effective manufacturing processes may provide an entry point for incorporating modified carbon nanotubes into our water remediation system [24]. Recent research indicates that it is possible to mass-produce high-quality modified carbon nanotubes at a reasonable cost. Numerous businesses are convinced that large-scale manufacturing at low pricing could serve as a gateway to replacing our current water treatment processes. According to the literature, the functionalization of carbon nanotubes with carboxyl and hydroxyl groups may apply to the photocatalytic oxidation of organic contaminants [25] (Figure 2). It is considered that carbon nanotubes still allow for significant and in-depth investigation of their electrical and lattice structures in order to fully comprehend the effect of functionalization bonding on carbon nanotubes. Prior to adopting large-scale applications, it is necessary to analyze the danger to the environment and human health. One of the key benefits of this approach is that covalent bonding between the functionalized carbon nanotubes and the polymer matrix is possible, resulting in significantly developed mechanical characteristics of composites via strong interfacial bonds. This process frequently achieves greater filler dispersion than melt mixing, and in the case of in situ polymerization, it is possible to separate aggregated nanotubes with little energy input using a low viscosity solvent such as toluene as a pretreatment solvent [26]. Heavy metal adsorption is demonstrated using functionalized derivatives of carbon nanotubes and graphene with large surface areas and adsorption sites, addressing the pressing heavy metal pollution problem. This critical study covers the synthesis and characterization methods of functionalized carbon nanotubes and graphene, as well as their applications in heavy metal adsorption, the impact of water chemistry on adsorption capacity, and the decontamination mechanism [27].

According to Khan et al., CNTs exhibit excellent characteristics and are thus considered major prospects for various applications in nanotechnology [29]. They have several disadvantages, the most significant of which are their insolubility and handling complexity [11]. The report summarizes the various research studies conducted so far on cementitious materials and discusses the present state-of-the-art and possible future directions for these composites [30]. Cwirzen et al. used sonication to functionalize MWCNTS and cover them with polyacrylic acid polymeric surfactants [31]. He found a homogenous dispersion, good workability, and a nearly 50% upsurge in the compressive strength of cement by adding the treated CNTs in very modest amounts (0.045–0.015 weight percent of cement). Again, it was discovered that a strong matrix-CNT adhesion is the result of covalent connections generated between CNT functional groups (hydroxyl) and cement matrix products. The low percentage of carbon nanotubes employed is advantageous in terms of material cost. Konsta-Gdoutos et al. found dispersion of MWCNTs applied in trace levels but did not believe that functionalizing CNTs was necessary [32]. CNTs were dispersed in water using a commercial surfactant and ultrasonication. Only 0.048 weight percent CNTs were added to the cement, resulting in a 45 percent increase in Young’s modulus and a 25% upsurge flexural strength. According to the scientists, adding carbon nanotubes to cement enhanced the fraction of high-stiffness C-S-H phases relative to low-stiffness, porous C-S-H phases and lowered total porosity.

Pitroda and Jethwa affirm that when it comes to developing high-performance, multifunctional, and ideal (high strength, flexibility, crack-free, and durable) construction materials, carbon nanotubes demonstrate promise for modifying or enhancing the properties of conventional construction materials such as steel and concrete [33]. Without a doubt, carbon nanotubes are an exceptionally promising material that has the potential to enable significant advancements in an advanced generation of technology, electric equipment, and biofields. As a result, carbon nanotubes can be incorporated into future construction studies. Finally, several proof-of-concept scenarios are discussed in which CNTs can play a critical role in redefining the scope and capability of civil engineering in general. In particular, high-performance, multifunctional, and ideal (high strength, flexibility, crack-free, and durable) construction materials based on carbon nanotubes promise to modify or enhance the properties of conventional construction materials.

Yakovlev et al. published an article describing their study on changing old-style building resources such as cement concrete, anhydrite binders, silicate paint, and a liquid glass-based fire-retardant coating [34]. Modification occurred as a result of dispersions of MWCNTs uniformly distributed in binding matrices used to prepare materials, resulting in changes to the structure and properties of materials and products. The microstructure of cellular silicate concrete that has been changed using CNTs has been improved, and its strength has been increased by 30%. The structural study of the modified anhydrite binding matrix revealed that the addition of MWCNTs accelerated the anhydrite binder’s hydration and structure formation processes. By incorporating MWCNTs, the shape of newly formed formations in the anhydrite matrix is substantially altered. By covering silicate with CNT dispersion, electromagnetic radiation is absorbed by up to 70%. The addition of CNTs to fire-resistant compositions based on liquid glass improves the structure and efficacy of the intumesced protective covering when exposed to flame.

Lu et al. examined the impact of MWCNTs on concrete strength and durability [35]. They discovered that increasing the amount of functionalized multiwalled carbon nanotubes in concrete considerably reduced water absorption, hence increasing the durability and resistance to water of the concrete [36]. Yakovlev et al. described their research on altering conventional building materials such as cement concrete, anhydrite binders, silicate paint, and a liquid glass-based fire-retardant coating [34]. The modification that resulted from the uniform distribution of MWCNTs in the binding matrices was used to produce materials, resulting in changes to the structure and properties of materials and products. According to Siddique et al., CNTs are primarily composed of elemental carbon and consist of a curved graphene layer composed of a single layer of carbon atoms arranged in a honeycomb pattern that may contain varying amounts of metal impurities depending on the manufacturing process [37]. Carbon nanotubes’ compressive, tensile, modulus of elasticity, flexural, porosity, electrical conductivity, and autogenous shrinkage properties are examined.

Saifuddin et al. described CNTs as allotropes of carbon with a nanostructure capable of a length-to-diameter ratio greater than one million [38]. These cylindrical carbon molecules exhibit unique characteristics, making them potentially helpful in a variety of nanotechnology applications. CNTs, which are formed from the graphene sheet, have remarkable mechanical qualities such as high toughness and high elastic modulus and thus represent an exciting new material with the potential to revolutionize a new generation of gadgets, electric equipment, and bio-sectors. Recent research indicates that it is possible to mass-produce high-quality modified CNTs at a reasonable cost [39]. Numerous businesses are convinced that large-scale manufacturing at low pricing could serve as a gateway to replacing our current water treatment processes.

CNT can be modified to transport bioactive peptides, proteins, nucleic acids, and medications to cells and organs [40]. Biocompatibility of carbon cylinders has been determined. It was crucial to evaluate the f-solubility of carbon nanotubes in physiological fluids since pure carbon nanotubes are extremely hazardous due to their insolubility. Second, appropriately functionalized carbon nanotubes appear to have a high predisposition for traversing cell membranes. CNT can also be loaded with biologically active molecules and delivered to a cell’s cytoplasm or nucleus. Due to the general chemistry of CNT, several functions, such as targeting molecules, contrast agents, medications, and reporter molecules, can be used on the same tube at the same time. Functionalized carbon nanotubes offer significant potential in nanotechnologies and biomedicine because they are non-immunogenic and have low toxicity [40].

## 3. Commercial Applications of Carbon Nanotubes

According to Bu et al., it is critical for the practical application of printed electronics to be able to print fully integrated energy storage components (for example, supercapacitors) [41]. Because printable materials are scarce, the majority of research on printed supercapacitors focuses on printing the electrode materials rather than the entire cell. This article details the first time a fully packed single-wall carbon nanotube-based supercapacitor was printed using direct ink writing (DIW) [42]. The article involves direct ink writing of fully-packaged supercapacitors on a flexible polymer substrate. DIW has used various supercapacitor designs, and electrochemical investigations indicate that a smaller gap between two electrodes and a broader electrode enables faster ion and electron transfer, respectively, which improves the total cell performance. A multi-cell supercapacitor array with three identical cells connected in series is printed to demonstrate the new technology’s scalability. The fully-packaged SW-CNT supercapacitor created via direct writing demonstrates comparable energy and power properties to recently reported carbon-based planer supercapacitors manufactured via different printing or non-printing procedures. The developed ink formula and direct ink writing setup enable mask-free, transfer-free, and alignment-free printing of various functional materials with precise and reproducible spatial distribution control. It enables the rapid, low-cost, and mass production of high-performance, fully-packaged products [42].

Carbon nanotube thin-film transistors are particularly well suited for controlling organic light-emitting diode (OLED) shows, as they exhibit higher mobility (approximately one cm^2^/V·s) and may be produced using low-temperature, non-vacuum processes [43]. In fuel cells, the use of carbon nanotubes as catalytic support can potentially cut Pt consumption by 60% when compared to carbon black [44], and doped carbon nanotubes may enable Pt-free fuel cells [45,46]. For organic solar cells, ongoing studies are aimed at utilizing the characteristics of carbon nanotubes to minimize undesirable carrier recombination and increase photo-oxidation resistance. In the long run, photovoltaic systems may combine CNT-Si heterojunctions and take advantage of the efficient creation of numerous excitons at p-n junctions generated within individual carbon nanotubes [47]. Mori et al. reveal the electrically driven ultrafast carbon nanotubes light emitter based on blackbody radiation with a response speed (110 Gbps) that is more than 106 times faster than conventional incandescent emitters and on par with or faster than light-emitting diodes or laser diodes [48]. This rapid response is explained by the CNT film’s unusually rapid temperature response, which is mostly due to the film’s limited heat capacity and high heat loss to the substrate. Additionally, we demonstrate experimentally a 140 ps width pulsed light generation and real-time optical communication. This carbon nanotube-based emitter provides novel topologies for optical interconnects, photonics, and optoelectronic integrated circuits due to its rapid response times, small footprint, and integration on silicon [48].

The adsorption of a variety of pharmaceuticals on various MWCNTs was used to investigate the possibility of MWCNTs as a carrier of new pharmacological contaminants. Diverse CNTs possessed different shapes, structures, and characteristics, which influenced their potential for adsorption of different medicinal chemicals, according to TEM observations. The Freundlich model was found to be better than the Langmuir model for adsorption isotherms. Multiple processes interacted to influence the adsorption behavior of different medications on CNTs, depending on the geometric forms, functional groups, and substituents of molecules. The change in charge would alter the electrostatic interaction between molecules and CNT surfaces by altering pH, resulting in a change in adsorption behavior. The adsorption of ionizable pharmaceuticals was significantly hampered by the salting-in effect, whereas for a non-ionized molecule such as carbamazepine, the effect was minor [49].

Tortorich and Choi observed that, while carbon nanotube inkjet printing is still in its infancy, it has enormous potential for a variety of uses, including flexible and printable electronics, transparent electrodes, and electronic sensors, due to its low cost and the extraordinary properties of CNTs [50]. Along with the composition of CNT ink and its printing procedures, this chapter discusses the most recent breakthroughs and achievements in CNT inkjet printing, as well as a quick summary of the technology’s prospects. Naturally, there are a few roadblocks to overcome before inkjet printing becomes a commercially viable method for depositing carbon nanotubes, but they will be soon addressed. Stable CNT inks will soon be commercially available as a result of current research in the field of CNT dispersion. Additionally, when compared to typical office inkjet printers, commercial inkjet printers such as the Fujifilm Dimatix offer higher control and resolution. Takagi et al. have even developed a way for further enhancing inkjet printing resolution by modifying the substrate surface [51]. P-type thin-film transistors were constructed using networks of pure semiconducting CNTs as the active material [29]. They were printed on flexible substrates such as polyimide [52] and polyethylene utilizing 3D printers equipped with inkjet or gravure heads (PET) [53], as well as clear substrates such as glass. These transistors display high mobility (>10 cm^2^/V·s), a high ON/OFF ratio (>1000), and a threshold voltage of less than 5 V. They provide a high current density and low power consumption, as well as environmental stability and mechanical adaptability [54]. The hysteresis of the current–voltage curses, as well as the volatility of the threshold voltage, remain unaddressed [55]. CNTs are a class of new class of nanomaterials with a nanometer diameter and a high length-to-diameter ratio. The nanotubes’ extraordinary mechanical and electrical capabilities are due to their quasi-one-dimensional (1D) structure and graphite-like arrangement of carbon atoms in the shells. Thus, the nanotubes have a high Young’s modulus and tensile strength, making them ideal for improving the mechanical properties of composite materials.

Tsentalovich, et al., 2017 found that a lightweight conductor that can replace copper can benefit a variety of industries, including automotive, portable electronics, and subsea power transmission [56]. The low density and incredible electrical properties of carbon nanotubes have been exploited to create a new generation of conductors in a growing number of research studies over the last decade. Drug-delivery devices that target the lymphatic system can effectively stop cancer spread. Radical polymerization can connect polyacrylic acid (PAA) to CNTs, making them highly hydrophilic. CNTs were only found in the local lymphatic nodes after 3 h of subcutaneous injection and not in the important organs such as the liver, kidney, heart, spleen, or lungs [56].

## 4. Uses of CNTs in Biomedicine

**Figure 3:**
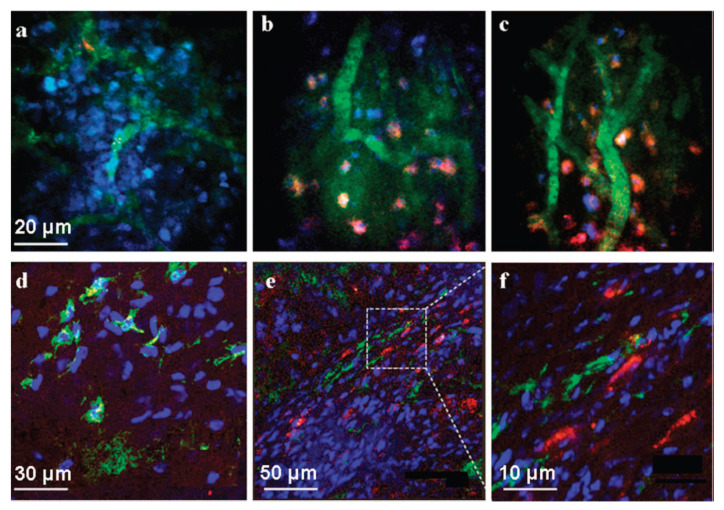
Detection of Nanotube Bioconjugates in Tumors in vivo. Representative frames from time-lapse videos acquired by 3-color, intravital two-photon microscopy (**a**–**c**). Mice bearing the HN12 xenografts were anesthetized and treated with SQ or SQE (red) bioconjugates. Cell nuclei were stained with Höechst (blue) and blood vessels with 500 kDa FITC-dextran (green): For SQ alone with no EGF (**a**), very little or no red fluorescence representing the Qdot signal was detected within the tumor mass 45 min after injection. Two different views after administration of SQE giving red fluorescence 45 min post injection within the tumor microenvironment (**b**,**c**). The red SQE bioconjugate is localized in close proximity to the nuclei suggesting its internalization by the tumor cells within the xenograft. (Scale bar in a-c is 20 µm). Confocal microscopy images of fixed xenograft cryosections (**d**–**f**) In the SQ treated tumor sections (**d**), only Höechst stained cell nuclei (blue) and vascular FITC-labeled dextran (green) are visible (scale bar 30 µm). (**e**) In SQE treated mice, characteristic red fluorescence was widely distribution within the tumor microenvironment. (scale bar 50 µm). (**f**) Magnified dotted region of (**e**) showing internalized SEQ bioconjugates the cells within the tumor mass. (scale bar 10 µm). (Reprinted with permission from [59]. Copyright 2009, Bhired et al.) (**a**–**f**).

Liu et al. coated branched polyethylene glycol (PEG)-functionalized SWNTs with doxorubicin (DOX) in order to prolong the period of blood circulation [57]. SWNT-DOX was injected into tumor-bearing mice. They demonstrated that doxorubicin can be directly injected into tumors and that single-walled nanotubes can be eliminated from the systemic blood circulation via the kidney. On the other hand, the physical loading of paclitaxel (PTX) is particularly difficult due to its limited solubility in an aqueous solution. Lay et al. addressed this barrier by synthesizing both PEG-grafted single-walled carbon nanotubes (PEG-gSWNTs) and PEG-grafted multiwalled carbon nanotubes (PEG-gMWNTs) (PEG-g MWNTs) [58]. Numerous novel carbon nanotube-based targeted therapies have been identified. Typically, these delivery systems comprise functional carbon nanotubes, targeted ligands, and an anticarcinogen (Figure 3). Carbon nanotubes can be used to include a variety of medications, polypeptides, and nucleonic acid due to their unusually large surface area. Due to endocytosis and other processes, functional carbon nanotubes can traverse the membrane of mammalian cells [59].

Carbon nanotubes have been researched as an immunotherapy for cancer. Tumor cell vaccines were utilized in this procedure, and inactivated cancer cells or dendritic cells displaying tumor antigens spurred the patient’s immune response against the tumor [60]. Recently, several studies suggested that heating carbon nanotubes put into cancer cells could induce their death. Photothermal therapy is a non-invasive, nontoxic, and very successful therapeutic procedure in which tumor tissues are heated locally using near-infrared (NIR) light, and then tumor cells are eliminated. CNTs exhibit excellent light absorption properties when exposed to near-infrared and radiofrequency radiation [61]. Additionally, the length of carbon nanotubes is critical since it determines their ability for heat transfer and cancer cell erosion [62]. Photothermal therapy systems based on carbon nanotubes can be combined with chemotherapy and gene therapy procedures to increase the efficiency of cancer treatment [63]. To increase the efficiency of tumor cell vaccines, the oxidized multiwalled carbon nanotubes can be covalently attached to the proteins in tumor lysate via an amide bond. On the other hand, Villa et al. demonstrated that SWCNTs could function as antigen-presenting carriers, hence augmenting the immune response to weak immunogenic peptides [64].

Cancian and colleagues demonstrated that scaffolds made of non-covalently attached CNTs in thermosensitive chitosan hydrogels had a suitable viscosity for local injection, a technique that might potentially replace invasive operations such as bone grafts [65]. Enhancing physical characteristics is especially advantageous for cardiac reinforcement applications, as electrical conductivity is required for both bone tissue regeneration and cardiac reinforcement. Numerous studies have demonstrated that the introduction of carbon nanotubes into biopolymer-based hydrogels promotes the formation of cardiomyocytes suitable for cardiac tissue engineering [66] or cardiac patches [67]. Additionally, Zhang et al. demonstrated that SWCNTs promote osteoblast proliferation in chitosan scaffolds containing crystalline hydroxyapatite and increased tensile and compressive modulus compared to pristine hydrogels [68]. Kaboudin et al. have revealed that nucleic acid can be transfected by a method other than endocytosis. MWCNTs functionalized with pyridine and magnetic particles transported nucleic acids bound by interactions over the cell membrane and into the cytoplasm [69]. The nanocarriers were subsequently removed from the cell using a magnetic field, thereby minimizing their cytotoxicity [70].

Magnetically assisted nanocarriers were also investigated by Xu et al. [71], who synthesized magnetic MWCNT in a single step using a paramagnetic surfactant coating. Electrostatic forces condense the DNA, allowing for endocytosis transfection of the nanoplatforms. Salts that acted as a barrier between the cargo and the CNTs allowed DNA to be released into the cytoplasm. Folkmann et al. [72] discovered that single-walled carbon nanotubes could cause oxidative damage to DNA in mice following oral gavage, while Fraczek et al. [73] discovered that single-walled carbon nanotubes and multiwalled carbon nanotubes implanted in the brain produce inflammation. Reza Eivazzadeh-Keihan et al. discovered that a wide variety of advanced biocompatible materials had been employed to create scaffolds for the formation of new bone [74]. Scaffolds made of carbon nanomaterials are ideal because they are biocompatible, mechanically stable, and readily available. They have a remarkable capacity for bone tissue regeneration, efficient cell proliferation, and osteogenic differentiation. Scaffolds are essentially templates for the growth, proliferation, regeneration, adhesion, and differentiation of bone stem cells, which are all critical processes in bone tissue creation. An appropriate scaffold should mimic the milieu seen in genuine bone. They describe a variety of carbon nanomaterials that can be utilized as scaffolds for bone tissue engineering, including graphene oxide (GO), carbon nanotubes, fullerenes, carbon dots (CDs), and nanodiamonds. The capabilities and critical importance of the family of carbon nanomaterial-based scaffolds are discussed in broad terms [74].

**Figure 4:**
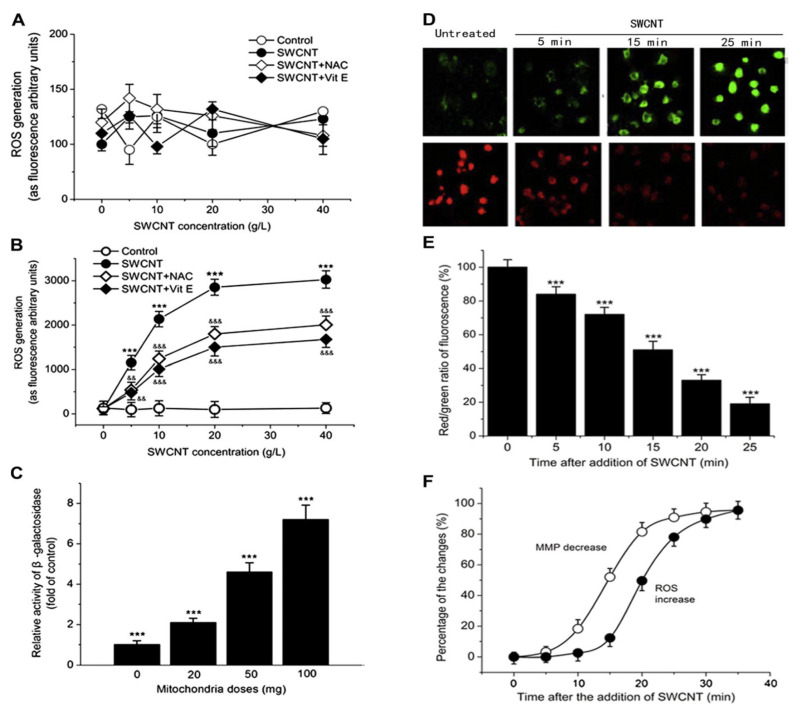
In vitro examination of the relationships between lysosomal and mitochondrial damage. (**A**) Effects of SWCNTs on the production of reactive oxygen species (ROS) in lysosomes. (**B**) Effects of SWCNTs on the production of ROS in mitochondria. NAC, N-acetylcysteine (10 μmol L^−1^); Vit E, vitamin E (10 μmol L^−1^). *** *p* < 0.001 compared with control. ^&&&^ *p* < 0.001 compared with SWCNT. ^&&^ *p* < 0.01 compared with SWCNT. (**C**) The influence of mitochondria on the lysosomal damage by SWCNTs. Mitochondria were added at the indicated concentrations, and the activities of β-galactosidase were measured. Lysosomal damage was expressed as the activity of the β-galactosidase leaked from lysosomes. *** *p* < 0.001 compared with control. (**D**) Fluorescence images showing the influences of SWCNTs on the mitochondrial membrane potential (MMP). Green fluorescence increased and red fluorescence decreased with incubation time. (**E**) Changes in the red-to-green fluorescence ratios in the mitochondrial membranes over 25 min of incubation with SWCNTs. SWCNTs significantly decreased the ratio time-dependently. (**F**) The time courses for the changes of both MMP and mitochondrial ROS production. (Reprinted with permission from [77]. Copyright 2010, Yang et al.).

The primary advantages of carbon nanomaterial-based scaffolds for bone tissue engineering are their significant growth stimulation, low cytotoxicity, efficient nutrient delivery within the scaffold microenvironment, appropriate functionalized chemical structures to facilitate cell–cell communication, and improvement of cell spreading [74,75]. Finally, research demonstrates that carbon nanoparticles are biodegradable in the bone tissue creation process. The biodegradation process can have an effect on cellular activity, nutrient exchange efficiency, and the cellular milieu on the scaffold surface, underlining the importance of high-quality preparation. Single-walled carbon nanotubes can be functionalized with antibodies against p-glycoprotein and loaded with the anticancer medication doxorubicin, as Li et al. demonstrated [76]. When compared to free doxorubicin, this formulation increased cytotoxicity against K562R leukemia cells by 2.4-fold. Yang et al. revealed that single-walled carbon nanotubes were effectively used to transport acetylcholine with a high safety margin in the brains of Alzheimer’s disease-affected mice [77]. Figure 4 describes numerous different functionalized single-walled carbon nanotubes or multiwalled carbon nanotubes that have been successfully used as vehicles for the delivery of drugs to treat neurodegenerative diseases or brain tumors [77].

## 5. Adsorption of Carbon Nanotubes

Carbon nanotubes exhibit remarkable adsorption capacity and effectiveness for the removal of lead from water [78]. The adsorption is strongly impacted by the solution’s pH and the nanotubes’ surface state, which can be modified during their processing. Both the Langmuir and Freundlich models adequately describe the adsorption isotherms. The results reveal that the adsorption characteristics of CNTs are strongly dependent on the pH of the solution [79]. The remarkable effectiveness of Pb^2^ removal by CNTs combined with their remarkable adsorption capacity shows that CNTs can be effective Pb^2^ adsorbers and have a wide range of potential uses in environmental protection [80]. Ma and colleagues have effectively developed triple-network CNTs/graphene oxide/sodium alginate nanocomposite hydrogels to remove antibiotics from contaminated wastewater [81] efficiently. The independent triple-network made use of its three-dimensional structure to enhance pollutant adsorption. Although the creation of double-network gels enhanced the internal structure, the three-dimensional space remained constrained and impossible to improve further. The hydrogel with a triple-network structure is a novel concept. Independent triple-network hydrogels feature bigger internal gaps and pores, as well as a more complex three-dimensional structure. In the future, composite processing of hydrogels, such as triple-network or multi-network hydrogels, will become a new technique for improving traditional hydrogels, emphasizing internal structure development rather than surface alteration [82].

Fiyadh et al. reviewed and examined the following topics: (a) the historical context of heavy metals in water and their effects on living organisms; (b) heavy metal remediation techniques, with a particular emphasis on adsorption; and (c) heavy metal removal using functionalized carbon nanotubes as adsorbents, most notably for the adsorption of the particularly hazardous metal arsenic [82]. Grace et al. studied MWCNT that had been manufactured on silicon wafers [83]. Iron particles (Fe) or combinations of iron–platinum (Fe-Pt) and iron–titanium (Fe-Ti) acted as catalysts for their growth. After non-direct and direct contact, the viability, growth, adhesion, and gene expression of L-929 and retinal precursor (R28) cells were investigated [84]. Non-direct contact had essentially no effect on cell development when compared to reference materials with known cytotoxicity levels. Both cell types proliferated well on each multiwalled carbon nanotube-coated wafer. The viability of the MWCNTs formed with the Fe-Pt and Fe-Ti catalyst combinations ranged from 95.9 to 99.8 percent, with non-functionalized MWCNTs surviving longer [85]. On MWCNT-coated wafers, R28 cells expressed less of the genes associated with neuronal and glial characteristics. Cell cycle proteins cyclin C (CCNC), MYC, and TP53 were all mildly downregulated. Cultivation on plasma-treated MWCNT resulted in no additional modifications. All MWCNT-coated slices examined demonstrated favorable biocompatibility profiles, indicating that this nanotechnology has the potential to significantly improve prostheses, including electrodes that connect to retinal tissue [86].

Two MWCNTs-Pd/Fe nanocomposites were used to evaluate the elimination of 2,4-dichlorophenol. The phenol adsorption efficiency of both materials was exceptional. Batch sorption experiments, including kinetics and isotherms, were also investigated systematically [87]. Zhou et al. investigated carbon-supported nZVI demonstrated rapid CAP removal with excellent capacity and electron efficiency. Despite the fact that the chemical compositions of AC-nZVI, BC-nZVI, CP-nZVI, CNTs-nZVI, and GO-nZVI were all the same, the distributions of nZVI particles varied depending on the carbon support, with the CP support being the most dispersive. These findings suggested that using carbon-supported nZVI to remediate CAP-contaminated water while also reducing antibiotic selection pressure in the environment could be a realistic option. 2,4 dichlorophenol (2,4-DCP) was thoroughly explored for its pathways, affecting variables, dechlorination kinetics, and selectivity [88].

Si, Shuxian et al. suggest a novel and effective technique for non-covalent modification of carbon nanotubes (CNTs) based on mussel-inspired polymerization of tannic acid (TA) and triethylenetetramine (TETA), followed by surface-initiated atom transfer radical polymerization (SI-ATRP). Fourier transform infrared spectroscopy (FT-IR), thermo-gravimetric analysis (TGA), transmission electron microscope (TEM), X-ray photoelectron spectroscopy (XPS), and photography were used to investigate the successful preparation of polymer brush grafted CNT (CNT-P(TA-TETA)-PDMAEMA) composite as well as the pH-responsive behavior of the composite [89].

Bankole et al. studied the synthesis, functionalization, and characterization of CNTs as batch adsorbents to remove selected heavy metals from industrial electroplating effluent [90]. The high-resolution scanning electron microscopy/high-resolution transmission electron microscopy/energy-dispersive X-ray spectrometer examination of the P-CNTs and PHB-CNTs indicated a well-defined morphology with a low degree of aggregation, whereas the Brunauer–Emmett–Teller (BET) analysis demonstrated considerable increases in the surface area and porosity of the CNTs following Poly(3-hydroxybutyrate (PHB) functionalization. Both nano adsorbents are widely polydispersed, with an aspect ratio in the range of 400–3200, as determined by DLS analysis. The presence of functional groups (C–C, C=C, C–OH, C=O, and O–C=O) and the presence of the –* bond were shown by XPS. Contact time, dosage, temperature, and pH all play a role in the batch adsorption process for removing heavy metals from electroplating effluent. The efficacy of the nano adsorbents in removing heavy metals is in the order of PHB-CNTs > P-CNTs, owing to the former’s higher surface area and presence of more functional groups. Ion exchange and electrostatic force adsorption methods are used in this investigation. The novelty of this study is that it established a one-way treatment approach (adsorption) for industrial electroplating wastewater utilizing newly created nano-adsorbents (P-CNTs and PHB-CNTs) with unmatched potential for and removal capabilities of heavy metals from wastewater. Overall, it was discovered for the first time that the adsorption behavior of both P-CNTs and PHB-CNTs is not just determined by their surface area but is also influenced indirectly by their water holding capacity and associated functionality. Finally, the treated adsorbate meets all applicable water quality standards for reuse in industrial or agricultural (irrigation) applications [90]. In a study examining the utilization of carbon nanotubes functionalized with an amino thiol to remove mercury, the single-walled carbon nanotubes -SH obtained adsorption effectiveness of 91% with a fivefold increase in the amount of CNTs [91]. Another work used magnetite nanocomposite/thiol functionalized multiwalled carbon nanotubes in combination with mercaptopropyl triethoxysilane (MPTS) grafted onto the CNTs/Fe_3_O_4_ surface to form MPTS-CNTs/Fe_3_O_4_ nanocomposites for the removal of both Pb^2^ and Hg^2^. Their adsorption capacities at pH 6.5 were 65.40 mg/g for Pb^2^ and 65.52 mg/g for Hg^2^, respectively. Adsorption capacity increased with increasing pH value, with 6.5 mg/g being the best pH for maximal adsorption, due to competition between H and metal ions and the tendency of metal ions to hydrate M(OH)_2_ at higher pH values [92,93].

According to Long et al., carbon nanotubes showed much greater dioxin elimination effectiveness than activated carbon [94]. Our prior research established that CNTs are excellent fluoride adsorbers and outperform activated carbon at removing fluoride. Duc Vu Quyen et al. effectively manufactured carbon nanotubes from LPG without the use of an initial hydrogen flow [95]. The characterization of the achieved carbon nanotubes reveals that the product is approximately 91.2 percent (*w*/*w*) hexagonal graphite pure. The tubes are multiwalled, lengthy, and less faulty, with an interior and external tube diameter of around 15 and 50 nm, respectively. The developed CNTs have a BET surface area of 134 m^2^/g. The tip-growth mechanism for the production of carbon nanotubes is proposed. The Fe_2_O_3_ precatalyst is reduced to iron, utilizing hydrogen and carbon produced during the breakdown of hydrocarbons. The surface of the produced carbon nanotubes was changed with potassium permanganate, and the resulting material had a high Cu (II) maximum adsorption capacity of 163.7 mg/g [95].

**Figure 5:**
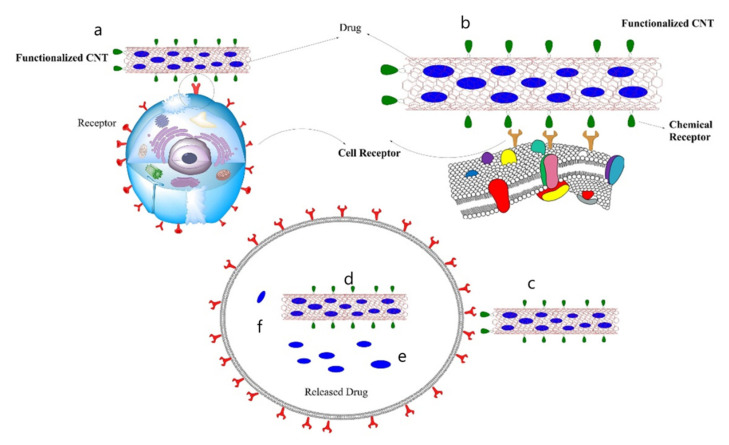
The drug delivery procedure is depicted graphically. (**a**) The surface of the carbon nanotube is linked to a chemical receptor and drugs are loaded inside, (**b**) the open end of the carbon nanotube is capped, (**c**) the drug-carbon nanotubes carrier is introduced into the body and reaches target cells via the chemical receptor on the carbon nanotubes surface, (**d**) the cell internalizes the carbon nanotubes via the endocytosis pathway, for example, (**e**,**f**) the cap is removed or biodegrades inside the cell, and then drugs are released.

The usage of zeolite carbon nanotubes (ZCNTs) as a novel approach for the removal of lead (2+) ions resulted in a 55.74 mg/g adsorption capacity. This ZCNTs kinetics model fits the Langmuir isotherm model to a pseudo-second-order degree [96]. Chen et al. described their work with multiwalled carbon nanotubes functionalized with KMnO_4_/H_2_SO_4_ and multiwalled carbon nanotubes functionalized with HNO_3_ [97]. The pseudo-second-order model provided excellent fitting to the experimental results, and the Elovich model revealed the adsorption mechanism to be chemisorption. Furthermore, the Langmuir equation best described the isotherm models, with functionalized MWCNTs having a greater adsorption capacity than virgin MWCNTs [97]. ABC transporters, a broad family of integral membrane proteins that inhibit drug absorption and accumulation in cells by active extrusion, are a primary cause of cancer multidrug resistance (MDR), which usually results in chemotherapy failure [98]. Carbon nanotube (CNT)-based drug delivery systems have considerable promise for improving cancer chemotherapy’s efficacy. However, the effects of carbon nanotubes on ATP-binding cassette (ABC) transporters remain unknown. MWCNTs have been found to inhibit the ABC transporter via a decrease in c-Myc expression. Oxidative stress does not appear to have a significant role in the mechanism behind this impact. This insight will help us better understand the biological impacts of carbon nanotubes and may aid in developing novel anticancer therapies, particularly for overcoming ABC transporter-mediated chemo-resistance. However, additional in-depth mechanistic investigations are required to completely clarify the activities of MWCNTs on Myc and ABC transporters and investigate their potential therapeutic benefits (Figure 5).

## 6. Silicon Chips with Carbon Nanotubes

A carbon nanotubes-based biosensor system-on-a-chip that integrates single-walled carbon nanotube-based biosensors and complementary metal oxide semiconductor-based signal processing circuitry is shown in [99]. This implementation was made possible by having to overcome a number of technological obstacles, including the design of an efficient signal processing approach, the assembly of uniform carbon nanotube devices using the linker-free directed assembly procedure, and the development of a biocompatible substrate functionalization strategy. This work is a significant step toward integrating 64 carbon nanotube-based sensors with complementary metal-oxide-semiconductor circuits. It will pave the way for a wide range of biomedical uses, such as sensing components in LoC systems for neuronal development culture and implantable real-time monitoring devices [99]. Fonverne et al. developed a silicon column for liquid chromatography comprised of carbon nanotube-coated micropillars [100]. Increased retention was observed when compared to a reference column coated with octadecyltrimethoxysilane (C-18), demonstrating the utility of carbon nanotubes as a stationary phase for chemical separations. Kim et al. reported the development of a novel gaseous formaldehyde sensing chip based on structured SWCT field-effect transistors and a carefully regulated aqueous layer with photopolymerized polyelectrolytic gels [101]. This method detects formaldehyde gas at a level of 0.1 parts per billion reliably, implying the development of a new type of indoor air quality monitoring instrument. This integrated chip is energy efficient and does not require any huge or expensive auxiliary devices. The suggested new type of gas sensing method has significant promise for practical applications such as freshness monitoring of items in refrigerators or warehouses by detecting water-soluble amine gases. Close et al. constructed an experimental prototype integrated circuit with high-speed CNT interconnects [102]. Developing nanotubes opens up new possibilities in fundamental science and real-world applications. The main goal is to use simple and effective methods to create defect-free nanotubes at the ton level. CNTs have a number of substantial technological challenges that will limit their widespread application in future devices. Most of these challenges are more application-specific; for example, controlled development and positioning of CNTs in transistor applications is a key challenge [103]. However, recent experimental development has shown that high-quality CNTs may be made at a much lower and more promising cost [104]. The purpose of this paper is to describe the manufacture of the world’s first stand-alone integrated circuit that combines silicon transistors and individual interconnected carbon nanotube wires on a single chip working at frequencies more than 1 GHz. Apart from reaching a milestone by working at frequencies more than 1 GHz, this prototype also serves as a platform for investigating carbon nanotubes at high frequencies on a silicon-based platform, opening the way for future multi-GHz nanoelectronics.

## 7. Conclusions

The global commercial demand for CNTs is evident in the fact that the industry currently produces several thousand tons each year. Extensive studies in the field of nanotoxicology, as well as rigorous government regulations, are required for identifying and avoiding toxic nanoparticles. Numerous countries have developed restrictions and laws to mitigate or avoid the possible risks associated with engineered nanoparticles in consumer items. This study provides an overview of carbon nanotubes in biomedical applications, emphasizing the numerous aspects and mechanisms impacting their toxicity. We reviewed numerous factors of carbon nanotubes, such as their size, length, agglomeration, and impurities that may contribute to oxidative stress, which is frequently the primary mechanism of their toxicity, making the micro/nanopatterns and architectures required for scalable devices made from CNTs a major challenge. According to the available literature, it can be understood that biomedical applications of CNTs have been progressing rapidly. It can be considered a promising biomedical agent for targeting, drug delivery, imaging, and sensors, etc., when compared with other nanomaterial’s still in use. The reviews mainly examine the recent developments of innovative CNT patterning, potential approaches, and the obstacles that lie ahead for future devices and applications.

## Data Availability

Not applicable.
